# Protein Tyrosine Kinase 7 (PTK7) in Breast Cancer: A Retrospective Analysis of Tumour Expression and Association with Clinical Outcome

**DOI:** 10.3390/cancers16183206

**Published:** 2024-09-20

**Authors:** Kate Lacey, Megan R. Greener, Tangkam R. Marak, Emad A. Rakha, Andrew R. Green, Ian O. Ellis, Stewart G. Martin, Sarah J. Storr

**Affiliations:** Nottingham Breast Cancer Research Centre, School of Medicine, University of Nottingham Biodiscovery Institute, University Park, Nottingham NG7 2RD, UKmegan.greener@nottingham.ac.uk (M.R.G.); tangkam.marak@nottingham.ac.uk (T.R.M.); emad.rakha@nottingham.ac.uk (E.A.R.); andrew.green@nottingham.ac.uk (A.R.G.); ian.ellis@nottingham.ac.uk (I.O.E.); stewart.martin@nottingham.ac.uk (S.G.M.)

**Keywords:** breast cancer, protein tyrosine kinase 7, PTK7, triple negative, prognosis, biomarkers

## Abstract

**Simple Summary:**

Protein tyrosine kinase 7 (PTK7) is a conserved catalytically defective transmembrane receptor that is a potential therapeutic target in triple-negative breast cancer, ovarian cancer, and non-small cell lung cancer. The aim of this retrospective study was to determine if PTK7 expression levels in breast tumours were associated with the clinical outcome of breast cancer patients. We determined PTK7 expression in patient tumours using immunohistochemistry and assessed *PTK7* mRNA expression in the METABRIC and TCGA cohorts. We determined that PTK7 expression was not clearly linked with survival in unselected patient cohorts; however, it was associated with breast cancer-specific survival of patients with poor and moderate Nottingham Prognostic Index values.

**Abstract:**

Protein tyrosine kinase 7 (PTK7), originally known as colon carcinoma kinase (CCK4), is an evolutionary conserved, catalytically defective transmembrane receptor involved in Wnt signalling. PTK7 has been identified as a potential therapeutic target, and a PTK7 antibody drug conjugate (PF-06647020; cofetuzumab pelidotin) has been investigated in phase I clinical trials for triple-negative breast cancer, ovarian cancer, and non-small cell lung cancer. PTK7 protein expression was evaluated in 1136 early-stage invasive breast tumours by immunohistochemistry. In addition, *PTK7* mRNA expression in the METABRIC (n = 1980) and the TCGA breast cancer cohorts (n = 1082) was evaluated. Associations between PTK7 expression and clinicopathological criteria and patient outcome were determined. No association between PTK7 protein expression and breast cancer-specific survival was observed; however, *PTK7* mRNA expression in the METABRIC cohort was associated with breast cancer-specific survival (*p* < 0.001). PTK7 protein and mRNA expression were associated with breast cancer-specific survival of patients with a poor prognostic Nottingham Prognostic Index (NPI) and a moderate prognostic NPI, respectively. Taken together, these data indicate that PTK7 expression is associated with patient outcome in subgroups of breast cancer patients.

## 1. Introduction

Protein tyrosine kinase 7 (PTK7), originally known as colon carcinoma kinase (CCK4), is an evolutionary conserved, catalytically defective transmembrane receptor that induces signalling through heterodimerisation with other receptor tyrosine kinases. Initial research on PTK7 identified its role as a regulator of planar cell polarity [1], with subsequent evidence demonstrating a role in cellular migration and wound healing [2,3]. PTK7 also interacts with Wnt ligands and known Wnt receptors (reviewed in [4]). PTK7 is reportedly overexpressed in several solid tumour types, including breast and ovarian cancer, and evidence indicates its role in tumour progression and breast cancer cell behaviour. Triple-negative breast cancer is a subtype of breast cancer where cells lack oestrogen (ER) and progesterone receptors, as well as human epidermal growth factor receptor 2 (HER2). It accounts for approximately 15% of breast cancers. PTK7 promotes the proliferation and migration of triple-negative breast cancer cell lines [5]; conversely, knockdown of PTK7 decreases cell adhesion, migration, and invasion of triple-negative breast cancer cell lines [6]. PTK7 expression also modulates oncogenic signal transduction in breast cancer in vitro [5,6].

Evidence is accumulating that PTK7 may be a promising anti-tumour target. Initial studies demonstrated that PTK7 is enriched on tumour-initiating cells in triple-negative breast cancer, ovarian cancer, and non-small cell lung cancer, and a humanised anti-PTK7 monoclonal antibody and auristatin microtubule inhibitor conjugate (PF-06647020; cofetuzumab pelidotin) induces sustained tumour regression in vivo [7]. PF-06647020 has a manageable safety profile in triple-negative breast cancer, ovarian cancer, and non-small cell lung cancer patients who have received multiple prior lines of standard-of-care therapy, with encouraging preliminary data on anti-tumour activity [8]. A phase I safety trial on PF-06647020 + gedatolisib, a pan PI3K/mTOR inhibitor in metastatic triple-negative breast cancer, has also been reported, indicating that the combination was well tolerated [9]. PTK7-targetting chimeric antigen receptor modified T cells (CAR T) have also been investigated in vitro and in vivo [10].

PTK7 protein expression in breast cancer has been investigated in several small studies, although large variations in the clinical endpoints assessed result in conflicting observations. In a cohort of 133 triple-negative breast cancer patients, no association with disease-free or overall survival was observed [11]. In a cohort of 118 breast cancer patients, high expression of PTK7 in lymph node metastases was associated with disease-free survival [12]. In 280 tumours, no association between PTK7 expression and overall survival was observed; however, an association between PTK7 expression and overall survival was observed in patients with triple-negative disease (n = 49) [5]. High PTK7 expression has also been associated with disease-free survival of patients treated with adjuvant chemotherapy (n = 103) [13]. In addition to survival, associations between available clinicopathological data and PTK7 have been determined. High PTK7 expression has been shown to be associated with oestrogen receptor (ER)-positive tumours in one study of 79 invasive ductal carcinomas [14]. In another study, PTK7 expression was not associated with ER status in the tumour; however, high PTK7 expression in lymph node metastases was strongly associated with ER-negative tumours [12]. This study also investigated breast cancer cell lines to show that *PTK7* mRNA expression was increased in ER-negative cell lines when compared to ER-positive cell lines [12].

The objective of this study was to evaluate PTK7 mRNA and protein expression in large and comprehensively annotated breast cancer cohorts to determine associations with clinicopathological criteria and patient survival.

## 2. Methods

### 2.1. Western Blotting

Antibodies used for immunohistochemistry had specificity determined using Western blotting. Invitrogen Bolt mini systems with 4–12% Bis–Tris plus gels were used for gel electrophoresis. Bolt LDS sample buffer and Bolt sample reducing buffer were used to prepare breast cancer cell lysates for electrophoresis, with sample denatured by incubation at 100 °C for ten minutes. Proteins were transferred to nitrocellulose (Whatman, GE Healthcare, Chicago, IL, USA) using Bolt transfer buffer with 10% methanol. Nitrocellulose was blocked in 3% non-fat milk in phosphate buffered saline (PBS) supplemented with Tween-20 for one hour before incubation, with anti-beta-actin (Abcam AB8226, 1:1000, Boston, MA, USA) and anti-PTK7 (Invitrogen MA5-25774, 1:1000, Waltham, MA, USA) antibodies overnight at 4 °C. PTK7 polyclonal antibody was raised against full-length recombinant PTK7 produced in HEK293T cells. Secondary antibodies, donkey anti-rabbit immunoglobulin (Li-Cor 926-32213, 1:10,000, Lincoln, NE, USA) and donkey anti-mouse immunoglobulin (Li-Cor 926-68072, 1:10,000), were incubated for one hour prior to visualisation on an Odyssey FC Imager (Li-Cor) using Image Studio software (V4.1).

### 2.2. Patient Cohort and Immunohistochemistry

A total of 1136 tumours from early-stage, primary operable, invasive breast cancer patients treated at Nottingham University Hospitals between 1998 and 2006 were investigated for PTK7 expression. Immunohistochemistry was performed on tissue microarrays that comprised single 0.6 mm cores, taken from representative tumour areas; the tumour areas were selected by a specialist breast cancer histopathologist from hematoxylin- and eosin-stained sections that have been reported previously [15,16,17]. This study is reported according to REMARK criteria [18].

Tissue microarray sections, 4 µm in thickness, were deparaffinised and subsequently rehydrated in a sequential immersion process in xylene, ethanol, and water. The tissue was heated in a microwave for 10 min at 750 W, followed by 10 min at 450 W in 0.01 mol L^−1^ sodium citrate buffer (pH 6.0) for antigen retrieval. Novolink polymer detection kits (Leica, Wetzlar, Germany) were utilised for tissue staining and were used according to the manufacturer’s instructions. Anti-PTK7 antibody (Invitrogen MA5-25774, 1:100) was incubated on tissue for one hour at room temperature. The tissue was dehydrated in ethanol and fixed in xylene before tissue staining and mounting using DPX; the slides were scanned using a Nanozoomer digital pathology scanner (Hamamatsu Photonics, Hamamatsu, Japan) at 200× magnification.

Cytoplasmic PTK7 staining was assessed using a semi-quantitative immunohistochemical H-score; the percentage area of tumour cells, with a staining intensity of 0 to 3, represented none, weak, intermediate, and strong intensity staining, respectively. The final H-score for each tumour was assessed by multiplying the intensity score with the percentage of positive cells. More than 30% of scores were double assessed, with both scorers blinded to the clinical outcome and each other’s scores. Independent scorers have a single measure intraclass correlation coefficient of >0.850, indicating good concordance between assessors.

All patients whose tumours were included on the tissue microarray underwent breast-conserving surgery or mastectomy, which was decided based on disease characteristics or patient choice, followed by radiotherapy if indicated. Ethical approval for use of tissue was issued by the North West–Greater Manchester Central Research Ethics Committee under the title: Nottingham Health Science Biobank (NHSB), reference number 15/NW/0685. The Nottingham Prognostic Index (NPI) as well as the ER and menopausal status was used to determine if the patients receive systemic adjuvant treatment. The NPI is calculated using the size of the tumour, the number of involved lymph nodes, and the grade of the tumour. Patients with an NPI score of less than 3.4 did not receive adjuvant treatment, and patients with an NPI score of 3.4 and above were candidates for CMF combination chemotherapy (cyclophosphamide, methotrexate, and 5-fluorouracil) if they were ER-negative or pre-menopausal and hormonal therapy if they were ER-positive. No patients received trastuzumab. Breast cancer-specific survival was calculated as the time interval between primary surgery and death resultant from breast cancer. Metastasis-free survival was defined as the time interval between primary surgery and metastasis. Clinical information for the cohort can be found in Table 1.

### 2.3. Statistical Analyses

IBM SPSS software (version 28) was used for statistical analysis, with cases stratified according to breast cancer-specific survival using X-tile software for both protein and mRNA expression [19]. The association of PTK7 expression with clinicopathological variables was determined using the Pearson χ^2^ test of association. The Kaplan–Meier method was used to produce survival curves using the log-rank test to determine significance. Multivariate survival analysis was performed using the Cox’s proportional hazard method. *p* values less than 0.05 were considered statistically significant.

### 2.4. METABRIC Cohort

The METABRIC cohort has been detailed elsewhere [20]. Briefly, five facilities in the UK and Canada contributed breast tumours collected between 1977 and 2005; consent was obtained from the respective institutional review boards as detailed in the original publication. Patients who were ER-negative and had positive lymph nodes underwent adjuvant chemotherapy, while those who were ER-positive and/or with positive lymph nodes did not. Trastuzumab was not administered to patients who had HER2-overexpressing tumours. Breast cancer-specific survival was computed as the duration between primary surgery and death attributed to breast cancer. Data analysis was performed on mRNA expression z-scores relative to all samples (log microarray) downloaded from http://www.cbioportal.org [21,22,23]. Gene set enrichment analysis (GSEA) was performed using GSEA software (Broad Institute, Cambridge, MA, USA, Version 4.3.2), with samples divided by categorised *PTK7* expression levels (https://www.gsea-msigdb.org/gsea/index.jsp) and gene enrichment determined in the curated hallmarks of cancer gene sets (h.all.v2023.2.Hs.symbols.gmt) [24,25,26]. Clinical information for this cohort can be found in Table 2.

### 2.5. TCGA Breast Cancer Cohort

The TCGA breast cancer cohort has been described previously [27]. Tumour tissues used in the TCGA study were obtained from patients at contributing centres with informed consent according to their local institutional review boards. Data analysis was performed on mRNA expression z-scores relative to all diploids (log RNASeq V2 RSEM) downloaded from http://www.cbioportal.org.

## 3. Results

### 3.1. PTK7 Protein Expression in Invasive Breast Cancer

**Figure 1:**
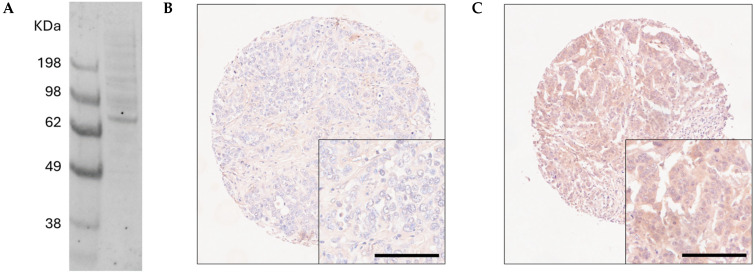
(**A**) Western blot assessment of PTK7 protein expression in MCF7 breast cancer cells. Representative photomicrographs of high PTK7 immunohistochemical staining (**B**) and low staining (**C**), where photomicrographs are shown at 10× magnification with 20× magnification inset box.

Antibody specificity was confirmed using Western blotting prior to staining patient tissues (Figure 1A). A total of 1136 breast cancer patient tumours were investigated for PTK7 expression. PTK7 demonstrated cytoplasmic staining with some granularity and heterogeneity between adjacent tumour cells, varying from weak to intense staining; very few cases with nuclear staining were observed, and representative staining is shown in Figure 1B,C. The median H-score for PTK7 was 120, ranging from 0 to 235. X-tile generated a cut point of 130, with 429 cases demonstrating a high PTK7 expression.

### 3.2. Associations between PTK7 Protein Expression and Clinicopathological Criteria

PTK7 protein expression was tested for association with clinicopathological criteria. High expression of PTK7 was significantly associated with lower grade (χ^2^ = 6.268, d.f. = 2, *p* = 0.044), higher NPI group (χ^2^ = 8.274, d.f. = 2, *p* = 0.016), ER-negative tumours (χ^2^ = 62.038, d.f. = 1, *p* < 0.001), PgR-negative tumours (χ^2^ = 144.180, d.f. = 1, *p* < 0.001), HER2-positive tumours (χ^2^ = 23.161, d.f. = 1, *p* < 0.001), and triple-negative tumours (χ^2^ = 26.109, d.f. = 1, *p* < 0.001). Standard NPI groupings used for assessment: good, moderate, and poor prognosis, defined by NPI value (<3.4, 3.5–5.4, and >5.4, respectively). No other significant associations between PTK7 protein expression and clinicopathological criteria were observed (Table 1).

**Table 1:** Associations between PTK7 expression and clinicopathological variables, determined using immunohistochemistry. The *p* values result from Pearson χ^2^ test of association, and significant values (*p* < 0.05) are highlighted in bold. ER is oestrogen receptor, and PgR is progesterone receptor.

		PTK7 Expression		
		Low	High	*p* Value
Tumour size				
	<2CM	445 (39.2%)	268 (23.6%)	0.873
	≥2CM	262 (23.1%)	161 (14.2%)	
Grade				
	1	122 (10.7%)	70 (6.2%)	**0.044**
	2	335 (29.5%)	176 (15.5%)	
	3	250 (22.0%)	183 (16.1%)	
Tubules				
	1	58 (5.1%)	35 (3.1%)	0.549
	2	224 (19.7%)	123 (10.8%)	
	3	425 (37.4%)	271 (23.9%)	
Pleomorphism				
	1	10 (0.9%)	9 (0.8%)	0.061
	2	254 (22.4%)	126 (11.1%)	
	3	443 (39.0%)	294 (25.9%)	
Mitosis				
	1	402 (35.4%)	219 (19.3%)	0.157
	2	134 (11.8%)	90 (7.9%)	
	3	171 (15.1%)	120 (10.6%)	
Lymphovascular invasion				
	Absent	506 (44.5%)	306 (26.9%)	0.930
	Present	201 (17.7%)	123 (10.8%)	
Lymph node status				
	Negative	414 (36.4%)	274 (24.1%)	0.076
	Positive	293 (25.8%)	155 (13.6%)	
NPI grouping				
	GPG	286 (25.2%)	152 (13.4%)	**0.016**
	MPG	300 (26.4%)	219 (19.3%)	
	PPG	121 (10.7%)	58 (5.1%)	
ER status				
	Negative	29 (2.6%)	78 (6.9%)	**<0.001**
	Positive	678 (59.7%)	351 (30.9%)	
PgR status				
	Negative	117 (10.4%)	212 (18.8%)	**<0.001**
	Positive	587 (52.1%)	210 (18.7%)	
HER2 status				
	Negative	666 (58.6%)	368 (32.4%)	**<0.001**
	Positive	41 (3.6%)	61 (5.4%)	
Triple-negative status				
	Non triple negative	685 (61.1%)	381 (34.0%)	**<0.001**
	Triple negative	17 1.5%)	21 (3.5%)	
Patient age				
	<50 years	214 (18.8%)	123 (10.8%)	0.568
	≥50 years	493 (43.4%)	306 (26.9%)	

### 3.3. Associations between PTK7 Protein Expression and Survival Outcome

**Figure 2:**
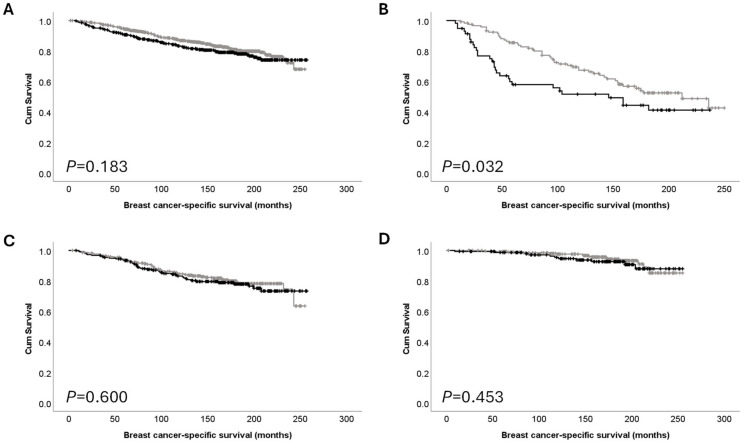
Kaplan–Meier analysis of breast cancer-specific survival showing the impact of low (grey line) and high (black line) protein expression: (**A**) PTK7 expression in the total cohort; (**B**) PTK7 expression in poor prognostic NPI group; (**C**) PTK7 expression in moderate NPI group; and (**D**) PTK7 expression in good NPI prognostic group.

Although PTK7 expression was not significantly associated with survival in the total patient cohort (*p* = 0.183) (Figure 2A), high expression of PTK7 was significantly associated with survival of those patients with poor NPI grouping (*p* = 0.032) (Figure 2B), but not medium or good NPI groups (*p* = 0.600 and *p* = 0.503, respectively) (Figure 2C,D). Other patient subgroups were also assessed, but no significant associations were identified.

### 3.4. Associations between PTK7 mRNA Expression and Clinicopathological Criteria

*PTK7* mRNA expression was analysed in the METABRIC patient cohort (n = 1980). Data analysis was performed on mRNA expression z-scores relative to all samples, with a median expression of −0.0756 ranging from −2.28 to 3.30. X-tile was used to generate a cut point to dichotomise the data into low and high expression. In total, 1000 cases showed low *PTK7* mRNA expression, and 980 cases showed high *PTK7* expression. *PTK7* mRNA expression was tested for association with clinicopathological criteria. High expression of *PTK7* mRNA was significantly associated with higher NPI group (χ^2^ = 38.023, d.f. = 2, *p* < 0.001), ER-negative tumours (χ^2^ = 203.406, d.f. = 1, *p* < 0.001), PgR-negative tumours (χ^2^ = 69.697, d.f. = 1, *p* < 0.001), and HER2-positive tumours (χ^2^ = 38.726, d.f. = 1, *p* < 0.001) (Table 2).

**Table 2:** Associations between mRNA expression of *PTK7* and clinicopathological variables in the METABRIC patient cohort. The *p* values are resultant from Pearson χ2 test of association, and significant values (*p* < 0.05) are highlighted in bold. ER is oestrogen receptor, and PgR is progesterone receptor.

		*PTK7* mRNA Expression		
		Low	High	*p* Value
Tumour size				
	<2CM	427 (21.9%)	425 (21.8%)	0.774
	≥2CM	559 (28.6%)	542 (27.8%)	
NPI grouping				
	GPG	405 (20.5%)	275 (13.9%)	**<0.001**
	MPG	517 (26.1%)	584 (29.5%)	
	PPG	78 (3.9%)	121 (6.1%)	
ER status				
	Negative	104 (5.3%)	370 (18.7%)	**<0.001**
	Positive	896 (46.3%)	610 (30.8%)	
PgR status				
	Negative	382 (19.3%)	558 (28.2%)	**<0.001**
	Positive	618 (31.2%)	422 (21.3%)	
HER2 status				
	Negative	921 (46.5%)	812 (41.0%)	**<0.001**
	Positive	79 (4.0%)	168 (8.5%)	
Lymph node status				
	Negative	501 (26.3%)	492 (25.8%)	0.938
	Positive	458 (24.1%)	453 (23.8%)	

**Figure 3:**
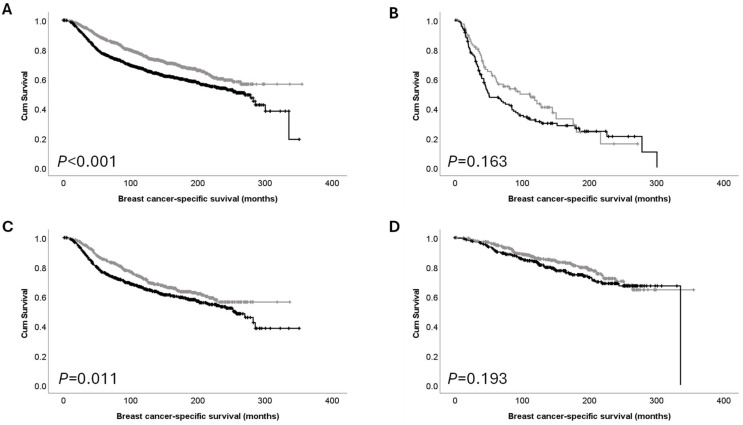
Kaplan–Meier analysis of breast cancer-specific survival showing the impact of low (grey line) and high (black line) mRNA expression: (**A**) *PTK7* mRNA expression in the total cohort; (**B**) *PTK7* mRNA expression in poor prognostic NPI group; (**C**) *PTK7* mRNA expression in moderate NPI group and (**D**) *PTK7* mRNA expression in good NPI prognostic group.

High *PTK7* mRNA expression was significantly associated with adverse survival of breast cancer patients (*p* < 0.001) (Figure 3A). No significant association was seen between *PTK7* mRNA expression and the survival of patients in the good and poor prognostic NPI groups (Figure 3B,D); there was, however, a significant association between high *PTK7* mRNA expression and the survival of patients in the moderate NPI group (*p* = 0.011) (Figure 3C).

*PTK7* mRNA expression was also analysed in the TCGA breast cancer patient cohort (n = 1082). Data analysis was performed on mRNA expression z-scores relative to diploid samples with a median expression of −0.0368, ranging from −1.52 to 13.24. X-tile was used to generate a cut point to dichotomise data into low and high expression, with 796 cases having low *PTK7* mRNA expression and 286 cases having high *PTK7* expression. *PTK7* mRNA expression was tested for association with clinicopathological criteria in this patient cohort. High expression of *PTK7* mRNA was significantly associated with the basal molecular subtype (χ^2^ = 187.093, d.f. = 4, *p* < 0.001) and the American Joint Committee on Cancer (AJCC) lymph node stage N0, assessed as N0, N1, N2, and N3 groups (χ^2^ = 7.936, d.f. = 3, *p* = 0.047). *PTK7* mRNA expression was not significantly associated with the AJCC tumour stage assessed as T1, T2, T3, and T4 groups (χ^2^ = 4.738, d.f. = 3, *p* = 0.192). High *PTK7* mRNA expression was not significantly associated with adverse survival of breast cancer patients in the TCGA cohort (*p* = 0.86). NPI groupings were not available for the TCGA cohort, so the validation of findings in these patient subgroups was not possible.

### 3.5. Associations between PTK7 Protein and mRNA Expression and the Survival of Patients with Receptor Negative/Positive Disease

**Figure 4:**
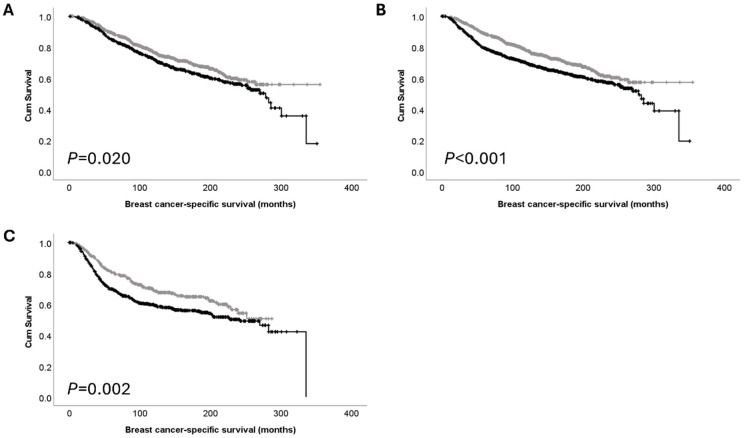
Kaplan–Meier analysis of breast cancer-specific survival showing the impact of low (grey line) and high (black line) mRNA expression: (**A**) *PTK7* mRNA expression in ER-positive subgroup; (**B**) *PTK7* mRNA expression in PgR-negative subgroup; (**C**) *PTK7* mRNA expression in HER2-negative subgroup.

Associations between *PTK7* protein and mRNA expression and the survival of patients with triple-negative disease have been reported previously and were therefore assessed in this large patient cohort. The METABRIC patient cohort was utilised for this assessment as no individual receptor data was available for the TCGA breast cancer cohort. No significant association between PTK7 protein expression and breast cancer-specific survival of ER-negative (*p* = 0.582), ER-positive (*p* = 0.836), PgR-negative (*p* = 0.651), PgR-positive (*p* = 0.997), HER2-negative (*p* = 0.060), or HER2-positive patients (*p* = 0.854) was observed. High *PTK7* mRNA protein expression was significantly associated with breast cancer-specific survival of ER-positive (*p* = 0.020), PgR-negative (*p* = 0.002), and HER2-negative patients (*p* < 0.001) (Figure 4A–C). No significant association between *PTK7* mRNA protein expression and the breast cancer-specific survival of ER-negative (*p* = 0.073), PgR-positive (*p* = 0.061), or HER2-positive patients (*p* = 0.528) was observed.

### 3.6. Gene Enrichment Analysis

GSEA was used to explore the METABRIC microarray data for enrichment of genes in the curated hallmarks of cancer gene sets in high- and low-*PTK7*-expressing tumours. The normalised enrichment scores (NES) with a false discovery rate of less than 1% identified 3/50 enriched gene sets in tumours with high expression of *PTK7* and 4/50 in tumours with low expression of *PTK7*. The gene sets enriched in high-*PTK7*-expressing tumours were HALLMARK MITOTIC SPINDLE (NES = 2.12, *p* < 0.001), HALLMARK APICAL JUNCTION (NES = 1.93, *p* < 0.001), and HALLMARK GLYCOLYSIS (NES = 1.74, *p* = 0.008). The gene sets enriched in low-*PTK7*-expressing tumours were HALLMARK ESTROGEN RESPONSE EARLY (NES = −1.99, *p* = 0.004), HALLMARK ESTROGEN RESPONSE LATE (NES = −1.84, *p* = 0.004), HALLMARK BILE ACID METABOLISM (NES = −1.71, *p* < 0.001), and HALLMARK FATTY ACID METABOLISM (NES = −1.70, *p* = 0.006).

## 4. Discussion

This study investigated *PTK7* mRNA and protein expression in large breast cancer cohorts (n = 1980, n = 1082, and n = 1136, respectively). We initially demonstrated that *PTK7* mRNA expression in the METABRIC cohort was associated with breast cancer-specific survival; however, this was not observed in the TCGA cohort or when protein expression was examined. We explored patient subgroups to determine if PTK7 expression was associated with breast cancer-specific survival and determined that protein and mRNA expression were associated with survival of poor prognostic NPI groups and moderate prognostic NPI groups, respectively. The research presented in this study demonstrates the largest investigation of PTK7 protein expression in primary breast tumours to date, although it is limited to tumours collected at one location within the United Kingdom.

There has been limited investigation of PTK7 in breast cancer, with studies focusing on patients with triple-negative disease. Previous research in a small number of triple-negative breast cancer patients (n = 133) did not show an association with patient survival; however, high levels of PTK7 were observed in smaller tumours [11]. A different study investigated 280 breast tumours. They did not observe an association with overall survival in the total patient cohort; however, an association was observed in patients with triple-negative disease (n = 49) [5]. In this study, similar results were found: no association with breast cancer-specific survival and PTK7 protein expression was observed, and no association between PTK7 protein expression and breast cancer-specific survival in receptor status subgroups was observed. We did, however, observe an association between *PTK7* mRNA expression and the survival of patients with ER-positive (*p* = 0.020), PgR-negative (*p* = 0.002), and HER2-negative tumours (*p* < 0.001) in the METABRIC cohort.

In addition to determining associations with survival, we also investigated associations with clinicopathological criteria. Previous research has demonstrated that high PTK7 expression was associated with ER-positive tumours in a cohort of 79 invasive ductal carcinomas, and that low PTK7 expression was associated with higher tumour grade and lymph node metastasis [14]. In this study, there were significant associations between high PTK7 protein expression and grade, higher NPI group, ER- or PgR-negative tumours, HER2-positive tumours, and triple-negative tumours. This was supported by the observation that high *PTK7* mRNA expression was significantly associated with higher NPI groups, ER- or PgR-negative tumours, and HER2-positive tumours in the METABRIC cohort. The same assessments were not possible in the TCGA breast cancer cohort. These significant associations partly support previously published studies that investigated patients with triple-negative disease; however, it may indicate that a subgroup of ER-negative/PgR-negative/HER2-positive patients may be of additional interest, particularly in light of studies investigating PTK7 as a therapeutic target in triple-negative breast cancer [7,8,9,10]. Interestingly, gene set enrichment analysis identified genesets that play a role in late and early oestrogen responses.

## 5. Conclusions

This study demonstrated that *PTK7* mRNA expression in the METABRIC patient cohort, but not PTK7 protein expression, is associated with breast cancer-specific survival. Conversely, *PTK7* mRNA expression in the TCGA patient cohort is not associated with breast cancer-specific survival. PTK7 protein and mRNA expression were associated with the survival of poor and moderate prognostic NPI groups, respectively. Perhaps unexpectedly, PTK7 protein expression was not associated with the survival of specific receptor status subgroups, although *PTK7* mRNA expression and the survival of patients with ER-positive, PgR-negative, and HER2-negative tumours were associated. These findings warrant further investigation in larger patient cohorts and indicate that PTK7 may be of clinical relevance in breast cancer.

## Data Availability

The mRNA expression data presented in this study are available for download (http://www.cbioportal.org), with immunohistochemistry data available on request.

## References

[1] Lu X., Borchers A.G., Jolicoeur C., Rayburn H., Baker J.C., Tessier-Lavigne M. (2004). PTK7/CCK-4 is a novel regulator of planar cell polarity in vertebrates. Nature.

[2] Lee H.K., Chauhan S.K., Kay E., Dana R. (2011). Flt-1 regulates vascular endothelial cell migration via a protein tyrosine kinase-7-dependent pathway. Blood.

[3] Shnitsar I., Borchers A. (2008). PTK7 recruits dsh to regulate neural crest migration. Development.

[4] Berger H., Wodarz A., Borchers A. (2017). PTK7 Faces the Wnt in Development and Disease. Front. Cell Dev. Biol..

[5] Cui N.P., Qiao S., Jiang S., Hu J.L., Wang T.T., Liu W.W., Qin Y., Wang Y.N., Zheng L.S., Zhang J.C. (2021). Protein Tyrosine Kinase 7 Regulates EGFR/Akt Signaling Pathway and Correlates With Malignant Progression in Triple-Negative Breast Cancer. Front. Oncol..

[6] Shin W.S., Oh S.W., Park H.N., Kim J.H., Lee S.T. (2023). Knockdown of PTK7 Reduces the Oncogenic Potential of Breast Cancer Cells by Impeding Receptor Tyrosine Kinase Signaling. Int. J. Mol. Sci..

[7] Damelin M., Bankovich A., Bernstein J., Lucas J., Chen L., Williams S., Park A., Aguilar J., Ernstoff E., Charati M. (2017). A PTK7-targeted antibody-drug conjugate reduces tumor-initiating cells and induces sustained tumor regressions. Sci. Transl. Med..

[8] Maitland M.L., Sachdev J.C., Sharma M.R., Moreno V., Boni V., Kummar S., Stringer-Reasor E., Lakhani N., Moreau A.R., Xuan D. (2021). First-in-Human Study of PF-06647020 (Cofetuzumab Pelidotin), an Antibody-Drug Conjugate Targeting Protein Tyrosine Kinase 7, in Advanced Solid Tumors. Clin. Cancer Res..

[9] Radovich M., Solzak J.P., Wang C.J., Hancock B.A., Badve S., Althouse S.K., Bray S.M., Storniolo A.M.V., Ballinger T.J., Schneider B.P. (2022). Initial Phase I Safety Study of Gedatolisib plus Cofetuzumab Pelidotin for Patients with Metastatic Triple-Negative Breast Cancer. Clin. Cancer Res..

[10] Jie Y., Liu G., Feng L., Li Y., Minyan E., Wu L., Li Y., Rong G., Li Y., Wei H. (2021). PTK7-Targeting CAR T-Cells for the Treatment of Lung Cancer and Other Malignancies. Front. Immunol..

[11] Ataseven B., Angerer R., Kates R., Gunesch A., Knyazev P., Hogel B., Becker C., Eiermann W., Harbeck N. (2013). PTK7 expression in triple-negative breast cancer. Anticancer Res..

[12] Gartner S., Gunesch A., Knyazeva T., Wolf P., Hogel B., Eiermann W., Ullrich A., Knyazev P., Ataseven B. (2014). PTK 7 is a transforming gene and prognostic marker for breast cancer and nodal metastasis involvement. PLoS ONE.

[13] Ataseven B., Gunesch A., Eiermann W., Kates R.E., Hogel B., Knyazev P., Ullrich A., Harbeck N. (2014). PTK7 as a potential prognostic and predictive marker of response to adjuvant chemotherapy in breast cancer patients, and resistance to anthracycline drugs. OncoTargets Ther..

[14] Sun J., Zhou Q., Tao Y., Chen J., Wang J. (2019). Loss of expression of protein tyrosine kinase 7 in invasive ductal breast cancers. Int. J. Clin. Exp. Pathol..

[15] Kotecha S., Lebot M.N., Sukkarn B., Ball G., Moseley P.M., Chan S.Y., Green A.R., Rakha E., Ellis I.O., Martin S.G. (2019). Dopamine and cAMP-regulated phosphoprotein 32 kDa (DARPP-32) and survival in breast cancer: A retrospective analysis of protein and mRNA expression. Sci. Rep..

[16] Saidy B., Kotecha S., Butler A., Rakha E.A., Ellis I.O., Green A.R., Martin S.G., Storr S.J. (2021). PP1, PKA and DARPP-32 in breast cancer: A retrospective assessment of protein and mRNA expression. J. Cell. Mol. Med..

[17] Saidy B., Vasan R., Durant R., Greener M.R., Immanuel A., Green A.R., Rakha E., Ellis I., Ball G., Martin S.G. (2023). Unravelling transcriptomic complexity in breast cancer through modulation of DARPP-32 expression and signalling pathways. Sci. Rep..

[18] McShane L.M., Altman D.G., Sauerbrei W., Taube S.E., Gion M., Clark G.M., for the Statistics Subcommittee of the NCI-EORTC Working Group on Cancer Diagnostics (2005). REporting recommendations for tumour MARKer prognostic studies (REMARK). Eur. J. Cancer.

[19] Camp R.L., Dolled-Filhart M., Rimm D.L. (2004). X-tile: A new bio-informatics tool for biomarker assessment and outcome-based cut-point optimization. Clin. Cancer Res..

[20] Curtis C., Shah S.P., Chin S.F., Turashvili G., Rueda O.M., Dunning M.J., Speed D., Lynch A.G., Samarajiwa S., Yuan Y. (2012). The genomic and transcriptomic architecture of 2000 breast tumours reveals novel subgroups. Nature.

[21] Cerami E., Gao J., Dogrusoz U., Gross B.E., Sumer S.O., Aksoy B.A., Jacobsen A., Byrne C.J., Heuer M.L., Larsson E. (2012). The cBio cancer genomics portal: An open platform for exploring multidimensional cancer genomics data. Cancer Discov..

[22] de Bruijn I., Kundra R., Mastrogiacomo B., Tran T.N., Sikina L., Mazor T., Li X., Ochoa A., Zhao G., Lai B. (2023). Analysis and Visualization of Longitudinal Genomic and Clinical Data from the AACR Project GENIE Biopharma Collaborative in cBioPortal. Cancer Res..

[23] Gao J., Aksoy B.A., Dogrusoz U., Dresdner G., Gross B., Sumer S.O., Sun Y., Jacobsen A., Sinha R., Larsson E. (2013). Integrative analysis of complex cancer genomics and clinical profiles using the cBioPortal. Sci. Signal..

[24] Liberzon A., Birger C., Thorvaldsdottir H., Ghandi M., Mesirov J.P., Tamayo P. (2015). The Molecular Signatures Database (MSigDB) hallmark gene set collection. Cell Syst..

[25] Liberzon A., Subramanian A., Pinchback R., Thorvaldsdottir H., Tamayo P., Mesirov J.P. (2011). Molecular signatures database (MSigDB) 3.0. Bioinformatics.

[26] Subramanian A., Tamayo P., Mootha V.K., Mukherjee S., Ebert B.L., Gillette M.A., Paulovich A., Pomeroy S.L., Golub T.R., Lander E.S. (2005). Gene set enrichment analysis: A knowledge-based approach for interpreting genome-wide expression profiles. Proc. Natl. Acad. Sci. USA.

[27] Hoadley K.A., Yau C., Hinoue T., Wolf D.M., Lazar A.J., Drill E., Shen R., Taylor A.M., Cherniack A.D., Thorsson V. (2018). Cell-of-Origin Patterns Dominate the Molecular Classification of 10,000 Tumors from 33 Types of Cancer. Cell.

